# Bile Salt Hydrolases: At the Crossroads of Microbiota and Human Health

**DOI:** 10.3390/microorganisms9061122

**Published:** 2021-05-22

**Authors:** Mélanie Bourgin, Aicha Kriaa, Héla Mkaouar, Vincent Mariaule, Amin Jablaoui, Emmanuelle Maguin, Moez Rhimi

**Affiliations:** 1 Institut Micalis, INRAE, Microbiota Interaction with Human and Animal Team (MIHA), AgroParisTech, Université Paris-Saclay, INRAE, 78350 Jouy-en-Josas, France; melbourgin@gmail.com (M.B.); aicha.kriaa@gmail.com (A.K.); hela.mkaouar@inrae.fr (H.M.); vincent.mariaule@inrae.fr (V.M.); amin.jablaoui@inrae.fr (A.J.); emmanuelle.maguin@inrae.fr (E.M.); 2 UPSP NP3 (2017.B146), Nutrition, Pathophysiology and Pharmacology, Oniris, College of Veterinary Medicine, Food Sciences and Engineering, Atlanpôle-La Chantrerie, Route de Gachet, 5 BP, 44300 Nantes, France

**Keywords:** bile salt hydrolases, bile acids, human health, gut microbiota, holobiont

## Abstract

The gut microbiota has been increasingly linked to metabolic health and disease over the last few decades. Several factors have been suggested to be involved in lipid metabolism and metabolic responses. One mediator that has gained great interest as a clinically important enzyme is bile salt hydrolase (BSH). BSH enzymes are widely distributed in human gastrointestinal microbial communities and are believed to play key roles in both microbial and host physiology. In this review, we discuss the current evidence related to the role of BSHs in health and provide useful insights that may pave the way for new therapeutic targets in human diseases.

## 1. Introduction

The gastrointestinal tract hosts a large and complex community of microorganisms, collectively known as the gut microbiota. This community consists of around 10^14^ microorganisms and is dominated by the presence of about 500–1000 different bacterial species [1,2]. The gut microbiota lives in a close relationship with the host and is crucial for many physiological processes [3,4]. Several mechanisms have been proposed to mediate the effects of these microorganisms on metabolic health. One important function carried out by the human gut microbiota is the deconjugation of primary bile acids (BAs) by BSH enzymes and their impact on host health [5,6]. The presence of active BSHs has long been recognized as a selection criterion for defining potential probiotics. BAs are amphipathic molecules that are initially produced by the host and subsequently transformed by the gut microbiota. Host primary BAs (CA, cholic acid; CDCA, chenodeoxycholic acid) are synthesized from cholesterol and conjugated with glycine or taurine in the liver (GCA and GCDCA, glycocholic acid and glycochenodeoxycholic acid; TCA and TCDCA, taurocholic acid and taurochenodeoxycholic acid). Conjugation improves the solubility of such hydrophobic BAs and reduces the potential damage of the cell membranes. Conjugated BAs are then excreted into the duodenum from the biliary duct, where they contribute to the solubilization of dietary lipids and their absorption through the small intestine and colon [7,8]. Around 95% of BAs are reabsorbed in the distal ileum. They are further effluxed into portal circulation, redirected, and stored in the liver through the enterohepatic cycle (EHC). The remaining primary BAs that escape EHC undergo further microbial biotransformations, including deconjugation, oxidation, epimerization, 7-dehydroxylation, esterification, and desulfatation by the gut microbiota [9]. The prominent secondary BAs are comprised of deoxycholic acid (DCA) and lithocholic acid (LCA), which result from the dehydroxylation of CA and CDCA, respectively [10,11]. Both primary and secondary BAs serve as signaling molecules that influence several processes, including lipid, glucose, and energy metabolism as well as inflammation [12,13].

Recently, increasing attention has been paid to better explore (i) the variety of enzymes catalyzing BA biotransformation, (ii) their distribution, and (iii) their relevance to the host’s health and to disease [14,15,16,17,18,19].

In this review, we discuss the current evidence related to microbial bile acid deconjugation, with a focus on gut bacterial bile salt hydrolases, and highlight its potential therapeutic interest.

## 2. Overview of Bile Salt Hydrolase Enzymes

Bile salt hydrolase (BSH; EC 3.5.1.24) is an enzyme produced by the intestinal microbiota that catalyzes the hydrolysis of amide bonds in conjugated BAs, resulting in the release of free amino acids [20]. These enzymes belong to the N-terminal nucleophilic (Ntn) hydrolase superfamily and share a similar αββα-core structure to an N-terminal catalytic cysteine residue [20]. This residue is critical to the catalysis mechanism and acts both as a nucleophile and a proton donor [21]. The N-terminal amino group serves as the proton acceptor and activates the nucleophilic thiol group of the cysteine side chain. Besides the cysteine residue, other amino acids conserved in most BSHs are also relevant to the catalytic reaction, including Arg18, Asp21, Asn82, Asn175, and Arg228 [20]. Numerous studies aimed to define the key amino acids and the secondary structural elements that are potentially involved in the substrate binding in BSHs [22,23]. These reports showed that BSHs are able to recognize their substrates via hydrophobic interactions with steroid moiety [24,25]. From the BSHs discovered, mainly intracellular enzymes were characterized as being from the *Bacteroides fragilis*, *Bacteroides vulgatus, Clostridium perfringens, Listeria monocytogenes, Lactobacillus,* and *Bifidobacterium* species [14]. Although most enzymes exhibited a similar overall topology, they displayed different catalytic efficiencies and substrate specificities. Additional biochemical reports suggested that around 58% of purified BSHs could recognize glyco-conjugated BAs [26]. To date, little data are available on the structural basis of BSH functions. Only five three-dimensional structures of the BSH enzymes from *Bifidobacterium longum* [27], *Lactobacillus salivarius* [23], *Enterococcus faecalis* [28], *Clostridium perfringens* [25], and *Bacteroides thetaiotaomicron* VPI-5482 [29] were reported. 

A deeper understanding of the structural features that contribute to substrate binding would offer further insights into the diverse preferences and functions of BSH enzymes.

## 3. Distribution of BSH-Active Bacteria in the Gut Microbiota

Many reports described that most studied BSHs are encoded by lactic acid bacteria and are used as probiotics to prevent mainly metabolic disorders in humans [30,31,32]. Recently, it was demonstrated that the human gut microbiota plays key a role in contributing to host health [33]. Among these microbial functions, the deconjugation of BAs by BSH enzymes comprises an imperative gateway reaction in the metabolism of BAs [6]. Considering the importance of BAs as biomolecules, gaps in our knowledge of these enzymes need to be filled. To uncover BSH gene abundance and their activity distribution in the gut microbiota, different approaches such as biochemical assays and metagenomic analysis are currently used; however, both approaches have inherent limitations. Indeed, standard biochemical assays that measure substrate consumption or product generation are unsuitable for defining and characterizing BSH enzymatic activities from complex biological samples. Through functional and comparative metagenomic analysis, Jones et al. highlighted the presence of BSH activity in all major bacterial divisions and the enrichment of BSHs in the human gut metagenomes of healthy subjects. Indeed, most metagenomic BSH-active clones were found to belong to *Firmicutes*, *Bacteroidetes,* and *Actinobacteria* [34]. Recently, taxonomic analyses among 11 different worldwide healthy populations have revealed 591 BSH enzymes, which are distributed over 117 genera and 447 bacterial strains [26]. The majority of the identified BSHs in the study belong to five genera, namely *Bacteroides*, *Blautia*, *Eubacterium*, *Clostridium,* and *Roseburia*. Around 27% of BSH-encoding bacteria behave as paralogs and are mainly assigned to *Bacteroides*. Given that the presence of paralogs introduces significant BSH sequence dissimilarity, Song et al. proposed an eight phylotype classification based on a phylogenetic tree as a more rational alternative than the classification by genera. A multivariable regression analysis showed that the relative abundance of BSHs does not significantly correlate with gender, age, and body mass index (BMI), while BSH distribution varies among populations from different geographical regions. At the biochemical level, enzyme activity assays highlighted different deconjugation selectivity between the phylotypes, in agreement with previous reports [26,35]. For example, while the T0 (*Clostridium*, *Intestinibacter*, *Lactobacillus*, *Enterococcus*), T1 (*Eubacterium*, *Blautia*, *Clostridium*, *Roseburia*, *Ruminococcus*), T3 (*Lactobacillus*), T4 (*Bifidobacterium* and *Collinsella*), and T7 (mainly *Blautia*) groups have significant specific activity for most bile salts, the T5 and T6 groups (assigned to *Bacteroides*) exhibit lower specificity towards GCA and both GCA/TCA, respectively. In contrast, the T1, T3, and T4 groups display the highest specific activities with GCA. When GCDCA and TCDCA are used as substrates, the highest specific activity is observed for group T2. Using a chemoproteomic approach to target BSH activities in the gut, Parasar et al. (2019) showed that this activity increases during intestinal inflammation, which results in higher deconjugated BAs [36]. Further studies are required to understand the relevance of gut BSH activities in host diseases.

## 4. BSH in Health and Disease

**Figure 1:**
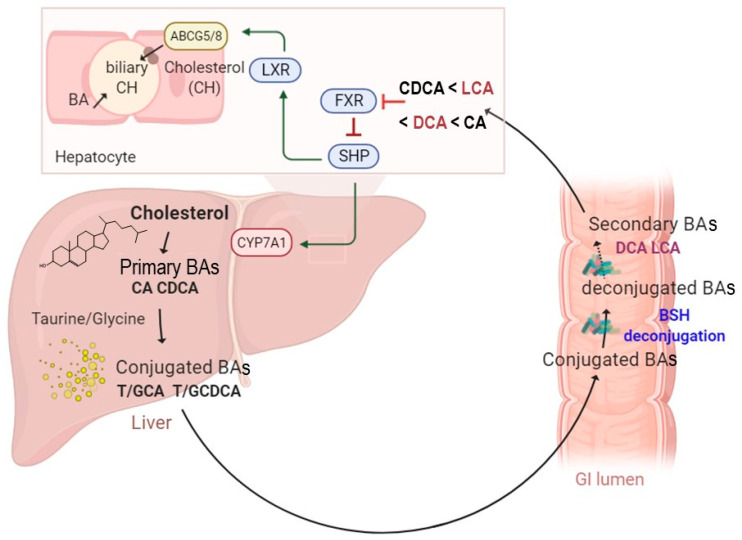
A schematic illustration of enterohepatic circulation and intestinal BA metabolism by BSH enzymes. The BAs are synthesized and conjugated with either taurine or glycine in the liver. They are further spilled over from the enterohepatic circulation and deconjugated by BSH-active gut bacteria. The hydrolysis of the conjugated BAs to deconjugated BAs and subsequent production of the secondary BAs can modulate several functions, including farnesoid X receptor (FXR), small heterodimer partner (SHP), cholesterol 7 alpha-hydroxylase (CYP7A1), liver X receptor (LXR), and ATP-binding cassette transporters G5/G8 heterodimer (*ABCG5/8*), which transports cholesterol into the bile canaliculi.

Several studies have investigated BSH-active bacteria for their clinical efficacy in lowering hypercholesterolemia [15,37,38]. These bacteria have been shown to increase BA deconjugation, which in turn decreases cholesterol absorption by enterocytes and enhances its fecal excretion [14]. Increased BSH activity was suggested to affect bile acid composition and to influence farnesoid X receptor (FXR) signaling. It has been demonstrated that reduced FXR activity promotes the downregulation of the small heterodimer partner (SHP) and increases the synthesis of BAs from cholesterol through the rate-limiting enzyme cholesterol 7α-hydroxylase (CYP7A1) [39] (Figure 1). The downregulation of the SHP elicits the activation of the liver X receptor (LXR), which in turn upregulates the adenosine triphosphate-binding cassette transporters G5 and G8 (ABCG5/G8) and promotes cholesterol transport into the bile [40,41] (Figure 1). The effect of BSH activity may be extended to weight gain through FXR signaling and the membrane receptor TGR-5. Previous studies demonstrated that the use of Tempol, an anti-oxidant, prevents obesity in mice through the reduction of BSH activity, resulting in FXR inhibition via the accumulation of tauro-β-muricholic acid. Furthermore, high-fat diet-fed FXR null mice showed reduced diet-induced obesity [12,42]. On the other hand, the activation of TGR-5 by BAs induced a significant weight reduction in mice on a high-fat diet. [43]. Obesity may also be associated with several liver-related diseases, including steatosis, hepatic cancer, and non-alcoholic fatty liver disease (NAFLD), which are linked to altered gut microbiota communities. NAFLD is a chronic liver disease with inflammation, hepatocyte injury, steatosis, and fibrosis as pathological features. Strategies for NAFLD treatment target the gut microbiota based on its ability to modulate insulin sensitivity and metabolism related to host energy, choline, and BAs. Of note, a clinical trial showed that VSL#3, a combination of eight probiotics, improves NAFLD through the increase in glucagon-like peptide-1 (GLP-1) [44]. Other probiotics, tested either in combination or isolated, also showed beneficial effects on NAFLD improvement [45,46]. Recently, Huang et al. demonstrated that *Lactobacillus plantarum* AR113 and *L. casei* pWQH0, which both display high BSH activity, decrease hepatic lipid accumulation in NAFLD cell models, which is potentially mediated by the BSH activity [47]. Besides cholesterol and lipid metabolism, BSH activity may be linked to inflammatory disorders. Gut metagenomic analysis revealed a reduced abundance in the BSH gene in patients suffering from inflammatory bowel diseases (IBD) and type 2 diabetes [48,49]. In the IBD context, the induced modification of the BA profile was suggested to alter the protective effect on the intestinal barrier mediated by FXR [49]. Repression of secondary BA metabolism via the modulation of BSH activity may represent a new therapeutic method for hepatic cancer treatment. In fact, primary BAs have been shown to stimulate the liver’s accumulation of hepatic NKT cells and antitumor immunity, thus inhibiting tumorigenesis in a mice model [50]. Besides host–microbiome crosstalk, BSHs are suggested to protect commensal bacteria from BA toxicity and to contribute to bacterial survival and gut colonization [51,52,53]. They are also believed to confer a nutritional advantage since released amino acids might be used as sources of nitrogen, carbon, and energy [14]. Altogether, these data stress the importance of BSH activity and BA receptors as therapeutic targets in numerous diseases.

## 5. Therapeutic Relevance on BSH-Active Bacteria

This global overview leads us to focus on the analysis of the relevant roles of BSHs on human health and their potential as therapeutic alternatives to treat human diseases.

### 5.1. Metabolic Disorders

Hypercholesterolemia is reflected by high plasma levels of both the total and LDL-cholesterol (LDL-c), while the HDL-cholesterol (HDL-c) levels decrease. High cholesterol levels are a major risk factor in the development of atherosclerosis and cardiovascular disease (CVD), the leading cause of mortality in the world [54]. Nowadays, BSH-active bacteria represent the main candidate for the prevention of hypercholesterolemia [14,55,56,57]. Several studies aiming to characterize the BSH encoding members of the gut microbiota were reported [34,35,58]. Not surprisingly, numerous potential probiotics with high BSH activity significantly reduce circulating cholesterol levels. This includes specific *Lactobacillus* and *bifidobacterial* species such as *B. longum*, *L. salivarius*, *L. plantarum,* and *L. reuteri* [16,37,56,59]. Interestingly, Sridevi et al. (2009) demonstrated that the oral administration of immobilized BSH from *Lactobacillus buchneri* significantly reduced both triglycerides and serum cholesterol using a rodent model of hypercholesterolemia [60]. Using a placebo-controlled, randomized study, Ooi et al. (2010) reported that the administration of a symbiotic capsule containing a BSH-active strain of *L. gasseri* with inulin resulted in significant reductions in total cholesterol and LDL-c compared to the placebo group [38]. Similar results were obtained with the *L. reuteri* strains. The effect of yogurts containing microencapsulated *L. reuteri* NCIMB 30242 was evaluated using a randomized, double-blinded study on hypercholesterolemic subjects. BSH-positive *Lactobacillus reuteri* NCIMB 30242 consumption over 6 and 9 weeks in [15,61], respectively, resulted in higher plasma deconjugated bile acid levels but lower total cholesterol, LDL-c, and non-HDL-c compared to the placebo group. Of note, *Lactobacillus reuteri* NCIMB30242 was the first strain of probiotics to be marketed (Cardioviva^®^) for cholesterol-reducing purposes based on BSH and the bile salt deconjugation mechanism. In another clinical trial, the consumption of yogurt containing *L. reuteri* CRL 1098 significantly decreased the total circulating cholesterol and LDL-c as well, while levels of HDL and triglycerides were unchanged [62]. Overall, these studies show that microbial BSH activity is able to impact lipid metabolism and reduce hypercholesterolemia. Most *Lactobacillus* species described as having a hypocholesterolemic effect in human trials have been patented [63,64,65,66]. The obtained hypocholesterolemic effects rely on the ability of BSH activity to deconjugate the primary intestinal BAs, thereby reducing cholesterol reabsorption [67]. 

In addition to cholesterol-lowering, accumulating research has been conducted to establish potential interplay between weight gain and/or obesity and gut microbiota-encoded BSHs through FXR signaling. Indeed, increasing evidence demonstrates that the microbiota, BAs, and FXR signaling are mandatory to induce obesity [12,68]. Additionally, the gut microbiota analysis of antibiotic-treated children revealed a correlation between body weight gain and prolonged reduction in overall BSH gene abundance [69]. In line with these findings, numerous BSH-expressing probiotics were shown to protect mice from weight gain and obesity as well as to influence the BA pool by modulating FXR signaling [70,71]. Advanced clinical studies showed that fecal microbiota transplantation in obese subjects did not reduce BMI but resulted in a BA profile similar to that of the donor [72,73]. Overall, these data support the hypothesis that targeted BSH enzymes can be delivered in order to control host lipidemia and to protect against weight gain. These effects may be particularly relevant seeing as some *Lactobacillus* and *Bifidobacterium* strains exhibiting BSH activity are regularly used in dairy products. In recognition of the importance of ensuring the host’s safety, these strains are commonly subjected to extensive characterization [74]. Furthermore, new therapeutic directions are focused on semi-synthetic FXR agonists, derived from the CDCA molecule [75]. Owing to the emergence of new BSH active probiotics, these strains can be tested to generate deconjugated BAs as FXR agonists. 

On the other hand, previous reports have suggested potential adverse effects of increased BSH activity in the intestine. High BSH activity is believed to generate significant amounts of unconjugated BAs, which may result in lipid malabsorption and trigger steatorrhea [76]. Increased BSH activity may also compromise lipid metabolism and colonic mucosal functions, thereby contributing to gallstone formation and colon cancer [77,78]. 

### 5.2. Infectious Diseases: Case of Giardia lamblia and Clostridium difficile

*Giardia duodenalis* (also named *G. lamblia* or *G. intestinalis*) is a protozoan parasite that infects humans and many animals through oro-fecal transmission [79,80,81]. *G. duodenalis* is widely distributed in the environment as a resistant cystic form; once ingested, these cysts differentiate into trophozoite (a flagelled replicative form) by excystation in the correct GI tract conditions [82]. The resulting enteropathogen can reach the colon and can cause giardiasis, one of the most common intestinal infectious diseases worldwide [83,84]. Considered a public health concern, several anti-infectious molecules have been developed to treat giardiasis [81]. However, the emergence of drug resistant strains limits the efficacy of the available treatments [81]. Recent reports established a strong interaction between *G. duodenalis* and the gut microbiota. In fact, it was highlighted that giardiasis susceptibility is highly influenced by the composition of the gut microbiota [85,86,87]. In turn, *G. duodenalis* colonization leads to gut microbiota composition changes [86]. Intriguingly, Riba et al. (2020) reported that *Giardia* infection alters the enteric microbiota composition in neonatal mice and modulates BAs and lipid metabolism, leading to reduced body weight gain and growth, as noted in infected infants [88]. Previous studies investigated the effect of probiotic bacteria (*L. johnsonii* La1, *L. gasseri* CNCM I-4884) in the field of giardiasis [89,90,91]. Both strains promote immune response, protect against *Giardia*-mediated tissue injury, and prevent trophozoite proliferation in murin models [89,91]. Of note, *L. gasseri* is more effective in vivo than *L. jonshoni* since *L. gasseri* reduces the cystic pool of the parasite [89]. Other lactobacillus strains, including *L. casei* MTCC 1423 and *L. rhamnosus,* have been tested in a murine model, showed similar protective effects, and reduced the severity/duration of giardiasis [92,93,94]. Interestingly, Shukla et al. (2020) reported an anti-giardia effect and enhanced mucosal immunity by the probiotic protein from *L. rhamnosus* as well as the heat-killed strain [95]. Although the underlying mechanism remains poorly understood [96,97,98], recent reports demonstrate a potential association between BSH activity and the protective effect against *G. duodenalis* infection [99,100]. As BSH activity allows for the conversion of primary BAs into deconjugated bile acids, it was suggested that the subsequent release of secondary BAs has a toxic effect and may alter parasite growth and survival. To address this hypothesis, the direct effects of conjugated and deconjugated BAs on the parasite were tested. Both tauro- and glyco-conjugated BAs were inert and did not inhibit parasite growth, while deconjugated BAs (DC and CDC) were toxic in a dose-dependent manner and exhibited a significant deleterious effect on the parasite [99]. Moreover, the simultaneous incubation of *G. duodenalis* with unconjugated BAs and BSH from *C. perfringens* allowed for the conversion of incubated BAs into their conjugated forms and consequently led to a killing effect on the parasite [99]. In addition, engineered *E. coli* to express BSH from *L. johnsonii* confirmed that BSH antagonized *Giardia* proliferation in vitro in a dose dependent manner as well as in vivo [100]. Altogether, these results support the promising prospect of using BSH active probiotics as a new therapeutic strategy to treat/prevent *G. dudenalis* infection.

Another example of a gastrointestinal infectious disease includes *Clostridioides difficile* infection (CDI). *C. difficile* is a spore-forming pathogen that might colonize the gut of patients treated by broad-spectrum antibiotics [101,102]. CDI includes a broad range of disorders ranging from diarrhea to colitis and toxic megacolon [101]. Their incidence, severity, and costs are in continuous increase. In addition, they have evolved as a significant cause of mortality during the past decade [101,103,104]. Upon germination, vegetative cells are generated from spores, and the bacterium produces toxins that result in tissues damage, inflammation, and diarrhea [105,106]. In the GI tract, the germination process is mediated by a host-derived molecule including BA sensing through the bile acid germinant receptor [105,107,108,109]. In fact, TCA, CA, and DCA activate germination, while CDCA acts as a competitive inhibitor [110]. Other studies indicate that DCA is a potent inhibitor of this process, as reported with *C. scindens*. Upon administration, this strain enhances the resistance to infection in a DCA dependent fashion [111]. Recent reports revealed that severe and relapsing CDI patients display high levels of TCA, followed by a reduced abundance of BSH-producing species [112,113]. However, fecal transplantation is effective in treating these patients and increases their resistance to CDI [112,113,114]. Further analysis demonstrates that, following FMT, key bacterial genera responsible for BSH production are recovered, BSH activity and BSH gene copy numbers are balanced, and secondary BAs are restored [112,113]. Interestingly, unlike patients pre-FMT, the BA profiles, similar to that in patients post-FMT, were able to inhibit spores outgrowth into vegetative forms [115]. In addition, supernatants from either engineered *E. coli* expressing highly active BSH or naturally BSH-producing organisms (*Bacteroides ovatus*, *Collinsella aerofaciens*, *Bacteroides vulgatus,* and *Blautia obeum*) effectively reduced TCA-mediated *C. difficile* germination. These effects were also confirmed in a recurrent CDI mouse model, using the recombinant *E. coli* strain for BSH [112]. These distinctive features pave the way towards the potential use of BSH active probiotics to treat CDI. However, active research must be conducted in this field to unravel the molecular mechanism of how secondary BAs may inhibit *C. difficile* outgrowth, colonization, and the subsequent inhibition of infection.

### 5.3. Other Human Pathologies

Several reports highlighted the association of many human disorders to BA dysregulation. In this context, we recently demonstrated the impact of the gut microbiota, cholesterol, and BA metabolism in hypercholesterolemia and cardiovascular diseases [116]. Furthermore, IBD, including ulcerative colitis and Crohn’s disease, are chronic immune-mediated inflammatory diseases that affect the gastrointestinal system. Targeted drugs are directed at the overactive immune response in these diseases [117]. Interestingly, several reports have suggested BA dysmetabolism as a contributing factor in IBD and irritable bowel syndrome (IBS) pathogenesis [118,119,120,121]. Among the potential mechanisms, the modification of the gut microbiota composition may lead to altered BA transformations in the gut and modulation of the circulating BAs. Direct enzyme activity assays in IBD patients revealed impaired deconjugation, dehydroxylation and, desulfation [122]. Otherwise, cholestasic diseases are also linked to changes in Bas, which are characterized by accumulated BAs levels in the biliary system. This dysfunction causes liver inflammation and injury [123]. 

Recently, the effect of ursodeoxycholic acid (UDCA) was studied in the context of the intrahepatic cholestasis of pregnancy (ICP) [124]. The reported results demonstrated that this BA counterbalances higher levels of BAs [124,125]. A recent study examined the clinical responses of the efficacy of the biotherapeutical agent UDCA in the case of the ICP cohort [124]. In this work, the authors were interested in the reaction mechanism associated with the effectiveness of UDCA, which counterbalances higher levels of BAs [124]. Such beneficial effects of UDCA were mediated by the gut microbiota through the enrichment of the BSH-expressing bacteria belonging to the *Bacteroidetes* phylum.

Modification of the BA metabolism is also linked to other human diseases such as liver disorders, including the primary sclerosing cholangitis (PSC). In this context, the gut microbiota and bile acid composition were analyzed in a mice model and demonstrated that the illness severity was increased in germ-free mice [126]. Moreover, Tabibian et al. (2016) described that PSC is reduced by the addition of UDCA, highlighting the protective potential of the gut microbiota and BA against PSC [126].

## 6. Conclusions

BSH enzymes play important roles in a wide range of host metabolic processes, including the regulation of cholesterol metabolism, energy, and inflammation homeostasis. BSHs have mainly been described in lactic acid bacteria. However, functional analysis of the gut microbiota revealed a high number of these enzymes in this ecological niche. The modulation of such activity has been shown to exhibit widespread effects on the host and resident microbiota. As the unique enzymes involved in the crucial deconjugation reaction, BSHs may serve as a promising strategy to control numerous diseases ranging from metabolic disorders to inflammatory and infectious diseases. Studies highlighting the molecular aspects of BSH enzymes including protein structure and genetic regulation would certainly be of great relevance to fully address and evaluate the implications of BSHs as a clinical tool.

## References

[1] Bäckhed F., Ley R.E., Sonnenburg J.L., Peterson D.A., Gordon J.I. (2005). Host-bacterial mutualism in the human intestine. Science.

[2] Gill S.R., Pop M., Deboy R.T., Eckburg P.B., Turnbaugh P.J., Samuel B.S., Gordon J.I., Relman D.A., Fraser-Liggett C.M., Nelson K.E. (2006). Metagenomic analysis of the human distal gut microbiome. Science.

[3] Marchesi J.R., Adams D.H., Fava F., Hermes G.D., Hirschfield G.M., Hold G., Quraishi M.N., Kinross J., Smidt H., Tuohy K.M. (2016). The gut microbiota and host health: A new clinical frontier. Gut.

[4] Contijoch E.J., Britton G.J., Yang C., Mogno I., Li Z., Ng R., Llewellyn S.R., Hira S., Johnson C., Rabinowitz K.M. (2019). Gut microbiota density influences host physiology and is shaped by host and microbial factors. eLife.

[5] Geng W., Lin J. (2016). Bacterial bile salt hydrolase: An intestinal microbiome target for enhanced animal health. Anim. Health Res. Rev..

[6] Foley M.H., O’Flaherty S., Barrangou R., Theriot C.M. (2019). Bile salt hydrolases: Gatekeepers of bile acid metabolism and host-microbiome crosstalk in the gastrointestinal tract. PLoS Pathog..

[7] Russell D.W. (2003). The enzymes, regulation, and genetics of bile acid synthesis. Annu. Rev. Biochem..

[8] Russell D.W. (2009). Fifty years of advances in bile acid synthesis and metabolism. J. Lipid Res..

[9] Ridlon J.M. (2020). Conceptualizing the Vertebrate Sterolbiome. Appl. Environ. Microbiol..

[10] Gérard P. (2013). Metabolism of cholesterol and bile acids by the gut microbiota. Pathogens.

[11] Ridlon J.M., Bajaj J.S. (2015). The human gut sterolbiome: Bile acid-microbiome endocrine aspects and therapeutics. Acta Pharm. Sin..

[12] Li F., Jiang C., Krausz K.W., Li Y., Albert I., Hao H., Fabre K.M., Mitchell J.B., Patterson A.D., Gonzalez F.J. (2013). Microbiome remodelling leads to inhibition of intestinal farnesoid X receptor signalling and decreased obesity. Nat. Commun..

[13] De Aguiar Vallim T.Q., Tarling E.J., Edwards P.A. (2013). Pleiotropic roles of bile acids in metabolism. Cell Metab..

[14] Begley M., Hill C., Gahan C.G. (2006). Bile salt hydrolase activity in probiotics. Appl. Environ. Microbiol..

[15] Jones M.L., Martoni C.J., Parent M., Prakash S. (2012). Cholesterol-lowering efficacy of a microencapsulated bile salt hydrolase-active *Lactobacillus reuteri* NCIMB 30242 yoghurt formulation in hypercholesterolaemic adults. Br. J. Nutr..

[16] Joyce S.A., MacSharry J., Casey P.G., Kinsella M., Murphy E.F., Shanahan F., Hill C., Gahan C.G. (2014). Regulation of host weight gain and lipid metabolism by bacterial bile acid modification in the gut. Proc. Natl. Acad. Sci. USA.

[17] Ridlon J.M., Harris S.C., Bhowmik S., Kang D.J., Hylemon P.B. (2016). Consequences of bile salt biotransformations by intestinal bacteria. Gut Microbes.

[18] Chand D., Avinash V.S., Yadav Y., Pundle A.V., Suresh C.G., Ramasamy S. (2017). Molecular features of bile salt hydrolases and relevance in human health. Biochim. Biophys. Acta Gen. Subj..

[19] Dong Z., Lee B.H. (2018). Bile salt hydrolases: Structure and function, substrate preference, and inhibitor development. Protein Sci..

[20] Ridlon J.M., Kang D.J., Hylemon P.B. (2006). Bile salt biotransformations by human intestinal bacteria. J. Lipid Res..

[21] Oinonen C., Rouvinen J. (2000). Structural comparison of Ntn-hydrolases. Protein Sci..

[22] Yadav R., Singh P.K., Puniya A.K., Shukla P. (2017). Catalytic interactions and molecular docking of bile salt hydrolase (BSH) from L. plantarum RYPR1 and its prebiotic utilization. Front. Microbiol..

[23] Xu F.Z., Guo F.F., Hu X.J., Lin J. (2016). Crystal structure of bile salt hydrolase from *Lactobacillus salivarius*. Acta Crystallogr. F Struct. Biol. Commun..

[24] Lambert J.M., Siezen R.J., De Vos W.M., Kleerebezem M. (2008). Improved annotation of conjugated bile acid hydrolase superfamily members in Gram-positive bacteria. Microbiology.

[25] Rossocha M., Schultz-Heienbrok R., von Moeller H., Coleman J.P., Saengers W. (2005). Conjugated bile acid hydrolase is a tetrameric N-terminal thiol hydrolase with specific recognition of its cholyl but not of its tauryl product. Biochemistry.

[26] Song Z., Cai Y., Lao X., Wang X., Lin X., Cui Y., Kalavagunta P.K., Liao J., Jin L., Shang J. (2019). Taxonomic profiling and populational patterns of bacterial bile salt hydrolase (BSH) genes based on worldwide human gut microbiome. Microbiome.

[27] Kumar R.S., Brannigan J.A., Prabhune A.A., Pundle A.V., Dodson G.G., Dodson E.J., Suresh C.G. (2006). Structural and functional analysis of a conjugated bile salt hydrolase from *Bifidobacterium longum* reveals an evolutionary relationship with penicillin V acylase. J. Biol. Chem..

[28] Chand D., Panigrahi P., Varshney N., Ramasamy S., Suresh C.G. (2018). Structure and function of a highly active Bile Salt Hydrolase (BSH) from *Enterococcus faecalis* and post-translational processing of BSH enzymes. Biochim. Biophys. Acta Proteins Proteom..

[29] Adhikari A.A., Seegar T.C.M., Ficarro S.B., McCurry M.D., Ramachandran D., Yao L., Devlin A.S. (2020). Development of a covalent inhibitor of gut bacterial bile salt hydrolases. Nat. Chem. Biol..

[30] Patel A.K., Singhania R.R., Pandey A., Chincholkar S.B. (2010). Probiotic bile salt hydrolase: Current developments and perspectives. Appl. Biochem. Biotechnol..

[31] Tsai C.C., Lin P.P., Hsieh Y.M., Zhang Z.Y., Wu H.C., Huang C.C. (2014). Cholesterol-lowering potentials of lactic acid bacteria based on bile-salt hydrolase activity and effect of potent strains on cholesterol metabolism in vitro and in vivo. Sci. World J..

[32] Tanaka H., Doesburg K., Iwasaki T., Mierau I. (1999). Screening of lactic acid bacteria for bile salt hydrolase activity. J. Dairy Sci..

[33] Cho I., Blaser M.J. (2012). The human microbiome: At the interface of health and disease. Nat. Rev. Genet..

[34] Jones B.V., Begley M., Hill C., Gahan C.G., Marchesi J.R. (2008). Functional and comparative metagenomic analysis of bile salt hydrolase activity in the human gut microbiome. Proc. Natl. Acad. Sci. USA.

[35] Yao L., Seaton S.C., Ndousse-Fetter S., Adhikari A.A., DiBenedetto N., Mina A.I., Banks A.S., Bry L., Devlin A.S. (2018). A selective gut bacterial bile salt hydrolase alters host metabolism. eLife.

[36] Parasar B., Zhou H., Xiao X., Shi Q., Brito I.L., Chang P.V. (2019). Chemoproteomic Profiling of Gut Microbiota-Associated Bile Salt Hydrolase Activity. ACS Cent. Sci..

[37] Damodharan K., Lee Y.S., Palaniyandi S.A., Yang S.H., Suh J.W. (2015). Preliminary probiotic and technological characterization of *Pediococcus pentosaceus* strain KID7 and in vivo assessment of its cholesterol-lowering activity. Front. Microbiol..

[38] Ooi L.G., Ahmad R., Yuen K.H., Liong M.T. (2010). *Lactobacillus gasseri* [corrected] CHO-220 and inulin reduced plasma total cholesterol and low-density lipoprotein cholesterol via alteration of lipid transporters. J. Dairy Sci..

[39] Thomas C., Pellicciari R., Pruzanski M., Auwerx J., Schoonjans K. (2008). Targeting bile-acid signalling for metabolic diseases. Nat. Rev. Drug Discov..

[40] Johnson B.J., Lee J.Y., Pickert A., Urbatsch I.L. (2010). Bile acids stimulate ATP hydrolysis in the purified cholesterol transporter ABCG5/G8. Biochemistry.

[41] Yoon H.S., Ju J.H., Kim H., Lee J., Park H.J., Ji Y., Shin H.K., Do M.S., Lee J.M., Holzapfel W. (2011). *Lactobacillus rhamnosus* BFE 5264 and *Lactobacillus plantarum* NR74 Promote Cholesterol Excretion Through the Up-Regulation of ABCG5/8 in Caco-2 Cells. Probiotics Antimicrob. Proteins.

[42] Gonzalez F.J., Jiang C., Patterson A.D. (2016). An Intestinal Microbiota-Farnesoid X Receptor Axis Modulates Metabolic Disease. Gastroenterology.

[43] Stanimirov B., Stankov K., Mikov M. (2015). Bile acid signaling through farnesoid X and TGR5 receptors in hepatobiliary and intestinal diseases. Hepatobiliary Pancreat. Dis. Int..

[44] Alisi A., Bedogni G., Baviera G., Giorgio V., Porro E., Paris C., Giammaria P., Reali L., Anania F., Nobili V. (2014). Randomised clinical trial: The beneficial effects of VSL#3 in obese children with non-alcoholic steatohepatitis. Aliment. Pharmacol. Ther..

[45] Famouri F., Shariat Z., Hashemipour M., Keikha M., Kelishadi R. (2017). Effects of Probiotics on Nonalcoholic Fatty Liver Disease in Obese Children and Adolescents. J. Pediatr. Gastroenterol. Nutr..

[46] Vajro P., Mandato C., Licenziati M.R., Franzese A., Vitale D.F., Lenta S., Caropreso M., Vallone G., Meli R. (2011). Effects of *Lactobacillus rhamnosus* strain GG in pediatric obesity-related liver disease. J. Pediatr. Gastroenterol. Nutr..

[47] Huang W., Wang G., Xia Y., Xiong Z., Ai L. (2020). Bile salt hydrolase-overexpressing *Lactobacillus* strains can improve hepatic lipid accumulation in vitro in an NAFLD cell model. Food Nutr. Res..

[48] Labbé A., Ganopolsky J.G., Martoni C.J., Prakash S., Jones M.L. (2014). Bacterial bile metabolising gene abundance in Crohn’s, ulcerative colitis and type 2 diabetes metagenomes. PLoS ONE.

[49] Ogilvie L.A., Jones B.V. (2012). Dysbiosis modulates capacity for bile acid modification in the gut microbiomes of patients with inflammatory bowel disease: A mechanism and marker of disease?. Gut.

[50] Ma C., Han M., Heinrich B., Fu Q., Zhang Q., Sandhu M., Agdashian D., Terabe M., Berzofsky J.A., Fako V. (2018). Gut microbiome-mediated bile acid metabolism regulates liver cancer via NKT cells. Science.

[51] De Smet I., Van Hoorde L., Vande Woestyne M., Christiaens H., Verstraete W. (1995). Significance of bile salt hydrolytic activities of lactobacilli. J. Appl. Bacteriol..

[52] De Boever P., Wouters R., Verschaeve L., Berckmans P., Schoeters G., Verstraete W. (2000). Protective effect of the bile salt hydrolase-active *Lactobacillus reuteri* against bile salt cytotoxicity. Appl. Microbiol. Biotechnol..

[53] Grill J.P., Cayuela C., Antoine J.M., Schneider F. (2000). Isolation and characterization of a *Lactobacillus amylovorus* mutant depleted in conjugated bile salt hydrolase activity: Relation between activity and bile salt resistance. J. Appl. Microbiol..

[54] Mendis S., Puska P., Norrving B., World Health Organization, World Heart Federation (2011). Global Atlas on Cardiovascular Disease Prevention and Control.

[55] Jones M.L., Tomaro-Duchesneau C., Martoni C.J., Prakash S. (2013). Cholesterol lowering with bile salt hydrolase-active probiotic bacteria, mechanism of action, clinical evidence, and future direction for heart health applications. Expert Opin. Biol. Ther..

[56] Wang G., Huang W., Xia Y., Xiong Z., Ai L. (2019). Cholesterol-lowering potentials of Lactobacillus strain overexpression of bile salt hydrolase on high cholesterol diet-induced hypercholesterolemic mice. Food Funct..

[57] Kriaa A., Bourgin M., Potiron A., Mkaouar H., Jablaoui A., Gérard P., Maguin E., Rhimi M. (2019). Microbial impact on cholesterol and bile acid metabolism: Current status and future prospects. J. Lipid Res..

[58] Yoon S., Yu J., McDowell A., Kim S.H., You H.J., Ko G. (2017). Bile salt hydrolase-mediated inhibitory effect of *Bacteroides ovatus* on growth of *Clostridium difficile*. J. Microbiol..

[59] Gu X.C., Luo X.G., Wang C.X., Ma D.Y., Wang Y., He Y.Y., Li W., Zhou H., Zhang T.C. (2014). Cloning and analysis of bile salt hydrolase genes from *Lactobacillus plantarum* CGMCC No. 8198. Biotechnol. Lett..

[60] Sridevi N., Vishwe P., Prabhune A. (2009). Hypocholesteremic effect of bile salt hydrolase from *Lactobacillus buchneri* ATCC 4005. Food Res. Int..

[61] Jones M.L., Martoni C.J., Prakash S. (2012). Cholesterol lowering and inhibition of sterol absorption by *Lactobacillus reuteri* NCIMB 30242: A randomized controlled trial. Eur. J. Clin. Nutr..

[62] Malpeli A., Taranto M., Cravero R., Tavella M., Fasano V., Vicentin D., Ferrari G., Magrini G., Hébert E., Valdez G. (2015). Effect of Daily Consumption of *Lactobacillus reuteri* CRL 1098 on Cholesterol Reduction in Hypercholesterolemic Subjects. Food Nutr. Sci..

[63] Prakash S., Jones M.L., Martoni C. (2020). Bacterial Compositions for Prophylaxis and Treatment of Degenerative Disease. U.S. Patent.

[64] Castellana J.C. (2014). Lactobacillus plantarum Strains as Hypocholesterolemic Agents. U.S. Patent.

[65] Zhen-Mei L., Mei-Gi H., Wen-Hsin L. (2011). Lactobacillus plantarum bb9 Capable of Adhering to Gastrointestinal Tract and Cholesterol Removal. U.S. Patent.

[66] Rothschild P., Connolly E., Möllstam B. (2008). Use of Selected Lactic Acid Bacteria for Reducing Atherosclerosis. U.S. Patent.

[67] Liong M.T., Shah N.P. (2005). Bile salt deconjugation ability, bile salt hydrolase activity and cholesterol co-precipitation ability of lactobacilli strains. Int. Dairy J..

[68] Parséus A., Sommer N., Sommer F., Caesar R., Molinaro A., Ståhlman M., Greiner T.U., Perkins R., Bäckhed F. (2017). Microbiota-induced obesity requires farnesoid X receptor. Gut.

[69] Korpela K., Salonen A., Virta L.J., Kekkonen R.A., Forslund K., Bork P., de Vos W.M. (2016). Intestinal microbiome is related to lifetime antibiotic use in Finnish pre-school children. Nat. Commun..

[70] Kim B., Park K.Y., Ji Y., Park S., Holzapfel W., Hyun C.K. (2016). Protective effects of *Lactobacillus rhamnosus* GG against dyslipidemia in high-fat diet-induced obese mice. Biochem. Biophys. Res. Commun..

[71] Degirolamo C., Rainaldi S., Bovenga F., Murzilli S., Moschetta A. (2014). Microbiota modification with probiotics induces hepatic bile acid synthesis via downregulation of the Fxr-Fgf15 axis in mice. Cell Rep..

[72] Zhang Z., Mocanu V., Cai C., Dang J., Slater L., Deehan E.C., Walter J., Madsen K.L. (2019). Impact of Fecal Microbiota Transplantation on Obesity and Metabolic Syndrome-A Systematic Review. Nutrients.

[73] Allegretti J.R., Kassam Z., Mullish B.H., Chiang A., Carrellas M., Hurtado J., Marchesi J.R., McDonald J., Pechlivanis A., Barker G.F. (2019). Effects of Fecal Microbiota Transplantation with Oral Capsules in Obese Patients. Clin. Gastroenterol. Hepatol..

[74] Jarocki P., Podleśny M., Glibowski P., Targoński Z. (2014). A new insight into the physiological role of bile salt hydrolase among intestinal bacteria from the genus *Bifidobacterium*. PLoS ONE.

[75] Han C.Y. (2018). Update on FXR Biology: Promising Therapeutic Target?. Int. J. Mol. Sci..

[76] Kim G., Lee B. (2005). Biochemical and Molecular Insights into Bile Salt Hydrolase in the Gastrointestinal Microflora—A Review. Anim. Biosci..

[77] Bongaerts G.P., Severijnen R.S., Tangerman A., Verrips A., Tolboom J.J. (2000). Bile acid deconjugation by *Lactobacilli* and its effects in patients with a short small bowel. J. Gastroenterol..

[78] Choi S.B., Lew L.C., Yeo S.K., Nair Parvathy S., Liong M.T. (2015). Probiotics and the BSH-related cholesterol lowering mechanism: A Jekyll and Hyde scenario. Crit. Rev. Biotechnol..

[79] Hooshyar H., Rostamkhani P., Arbabi M., Delavari M. (2019). Giardia lamblia infection: Review of current diagnostic strategies. Gastroenterol. Hepatol. Bed Bench.

[80] Thompson R.A., Monis P. (2012). *Giardia*—From genome to proteome. Adv. Parasitol..

[81] Gardner T.B., Hill D.R. (2001). Treatment of giardiasis. Clin. Microbiol. Rev..

[82] Ankarklev J., Jerlström-Hultqvist J., Ringqvist E., Troell K., Svärd S.G. (2010). Behind the smile: Cell biology and disease mechanisms of *Giardia* species. Nat. Rev. Microbiol..

[83] Thompson R.C., Reynoldson J.A., Mendis A.H. (1993). Giardia and giardiasis. Adv. Parasitol..

[84] Allain T., Amat C.B., Motta J.P., Manko A., Buret A.G. (2017). Interactions of *Giardia* sp. with the intestinal barrier: Epithelium, mucus, and microbiota. Tissue Barriers.

[85] Burgess S.L., Gilchrist C.A., Lynn T.C., Petri W.A. (2017). Parasitic Protozoa and Interactions with the Host Intestinal Microbiota. Infect. Immun..

[86] Barash N.R., Maloney J.G., Singer S.M., Dawson S.C. (2017). Giardia alters commensal microbial diversity throughout the murine gut. Infect. Immun..

[87] Torres M.F., Uetanabaro A.P.T., Costa A.F., Alves C.A., Farias L.M., Bambirra E.A., Penna F.J., Vieira E.C., Nicoli J.R. (2000). Influence of bacteria from the duodenal microbiota of patients with symptomatic giardiasis on the pathogenicity of *Giardia duodenalis* in gnotoxenic mice. J. Med. Microbiol..

[88] Riba A., Hassani K., Walker A., van Best N., von Zeschwitz D., Anslinger T., Sillner N., Rosenhain S., Eibach D., Maiga-Ascofaré O. (2020). Disturbed gut microbiota and bile homeostasis in *Giardia*-infected mice contributes to metabolic dysregulation and growth impairment. Sci. Transl. Med..

[89] Allain T., Chaouch S., Thomas M., Travers M.A., Valle I., Langella P., Grellier P., Polack B., Florent I., Bermúdez-Humarán L.G. (2018). Bile Salt Hydrolase Activities: A Novel Target to Screen Anti-*Giardia* Lactobacilli?. Front. Microbiol..

[90] Pérez P.F., Minnaard J., Rouvet M., Knabenhans C., Brassart D., De Antoni G.L., Schiffrin E.J. (2001). Inhibition of *Giardia intestinalis* by Extracellular Factors from Lactobacilli: An In Vitro Study. Appl. Environ. Microbiol..

[91] Humen M.A., De Antoni G.L., Benyacoub J., Costas M.E., Cardozo M.I., Kozubsky L., Saudan K.Y., Boenzli-Bruand A., Blum S., Schiffrin E.J. (2005). *Lactobacillus johnsonii* La1 antagonizes *Giardia intestinalis* in vivo. Infect. Immun..

[92] Shukla G., Devi P., Sehgal R. (2008). Effect of *Lactobacillus casei* as a probiotic on modulation of giardiasis. Dig. Dis. Sci..

[93] Shukla G., Sidhu R.K. (2011). *Lactobacillus casei* as a probiotic in malnourished *Giardia lamblia*-infected mice: A biochemical and histopathological study. Can. J. Microbiol..

[94] Goyal N., Rishi P., Shukla G. (2013). *Lactobacillus rhamnosus* GG antagonizes *Giardia intestinalis* induced oxidative stress and intestinal disaccharidases: An experimental study. World J. Microbiol. Biotechnol..

[95] Shukla G., Kamboj S., Sharma B. (2020). Comparative analysis of antigiardial potential of heat inactivated and probiotic protein of probiotic *Lactobacillus rhamnosus* GG in murine giardiasis. Probiotics Antimicrob. Proteins.

[96] Travers M.A., Florent I., Kohl L., Grellier P. (2011). Probiotics for the control of parasites: An overview. J. Parasitol. Res..

[97] Vitetta L., Saltzman E.T., Nikov T., Ibrahim I., Hall S. (2016). Modulating the Gut Micro-Environment in the Treatment of Intestinal Parasites. J. Clin. Med..

[98] Bustos A.Y., de Valdez G.F., Fadda S., Taranto M.P. (2018). New insights into bacterial bile resistance mechanisms: The role of bile salt hydrolase and its impact on human health. Food Res. Int..

[99] Travers M.A., Sow C., Zirah S., Deregnaucourt C., Chaouch S., Queiroz R.M., Charneau S., Allain T., Florent I., Grellier P. (2016). Deconjugated Bile Salts Produced by Extracellular Bile-Salt Hydrolase-Like Activities from the Probiotic *Lactobacillus johnsonii* La1 Inhibit *Giardia duodenalis* In vitro Growth. Front. Microbiol..

[100] Allain T., Chaouch S., Thomas M., Vallée I., Buret A.G., Langella P., Grellier P., Polack B., Bermúdez-Humarán L.G., Florent I. (2018). Bile-Salt-Hydrolases from the Probiotic Strain *Lactobacillus johnsonii* La1 Mediate Anti-giardial Activity in Vitro and in Vivo. Front. Microbiol..

[101] Smith A.B., Soto Ocana J., Zackular J.P. (2020). From Nursery to Nursing Home: Emerging Concepts in *Clostridioides difficile* Pathogenesis. Infect. Immun..

[102] Rupnik M., Wilcox M.H., Gerding D.N. (2009). *Clostridium difficile* infection: New developments in epidemiology and pathogenesis. Nat. Rev. Microbiol..

[103] Lessa F.C., Winston L.G., McDonald L.C., Emerging Infections Program *C. difficile* Surveillance Team (2015). Burden of *Clostridium difficile* infection in the United States. N. Engl. J. Med..

[104] Rineh A., Kelso M.J., Vatansever F., Tegos G.P., Hamblin M.R. (2014). *Clostridium difficile* infection: Molecular pathogenesis and novel therapeutics. Expert Rev. Anti Infect. Ther..

[105] Zhu D., Sorg J.A., Sun X. (2018). *Clostridioides difficile* Biology: Sporulation, Germination, and Corresponding Therapies for *C. difficile* Infection. Front. Cell. Infect. Microbiol..

[106] Abt M.C., McKenney P.T., Pamer E.G. (2016). *Clostridium difficile* colitis: Pathogenesis and host defence. Nat. Rev. Microbiol..

[107] Francis M.B., Sorg J.A. (2016). Dipicolinic Acid Release by Germinating *Clostridium difficile* Spores Occurs through a Mechanosensing Mechanism. mSphere.

[108] Francis M.B., Allen C.A., Shrestha R., Sorg J.A. (2013). Bile acid recognition by the *Clostridium difficile* germinant receptor, CspC, is important for establishing infection. PLoS Pathog..

[109] Francis M.B., Allen C.A., Sorg J.A. (2015). Spore cortex hydrolysis precedes dipicolinic acid release during *Clostridium difficile* spore germination. J. Bacteriol..

[110] Sorg J.A., Sonenshein A.L. (2008). Bile salts and glycine as cogerminants for *Clostridium difficile* spores. J. Bacteriol..

[111] Buffie C.G., Bucci V., Stein R.R., McKenney P.T., Ling L., Gobourne A., No D., Liu H., Kinnebrew M., Viale A. (2015). Precision microbiome reconstitution restores bile acid mediated resistance to *Clostridium difficile*. Nature.

[112] Mullish B.H., McDonald J., Pechlivanis A., Allegretti J.R., Kao D., Barker G.F., Kapila D., Petrof E.O., Joyce S.A., Gahan C. (2019). Microbial bile salt hydrolases mediate the efficacy of faecal microbiota transplant in the treatment of recurrent *Clostridioides difficile* infection. Gut.

[113] Weingarden A.R., Chen C., Bobr A., Yao D., Lu Y., Nelson V.M., Sadowsky M.J., Khoruts A. (2014). Microbiota transplantation restores normal fecal bile acid composition in recurrent *Clostridium difficile* infection. Am. J. Physiol. Gastrointest. Liver Physiol..

[114] van Nood E., Vrieze A., Nieuwdorp M., Fuentes S., Zoetendal E.G., de Vos W.M., Visser C.E., Kuijper E.J., Bartelsman J.F., Tijssen J.G. (2013). Duodenal infusion of donor feces for recurrent *Clostridium difficile*. N. Engl. J. Med..

[115] Weingarden A.R., Dosa P.I., DeWinter E., Steer C.J., Shaughnessy M.K., Johnson J.R., Khoruts A., Sadowsky M.J. (2016). Changes in Colonic Bile Acid Composition following Fecal Microbiota Transplantation Are Sufficient to Control *Clostridium difficile* Germination and Growth. PLoS ONE.

[116] Le Roy T., Lécuyer E., Chassaing B., Rhimi M., Lhomme M., Boudebbouze S., Ichou F., Haro Barceló J., Huby T., Guerin M. (2019). The intestinal microbiota regulates host cholesterol homeostasis. BMC Biol..

[117] Beaugerie L., Kirchgesner J. (2019). Balancing Benefit vs. Risk of Immunosuppressive Therapy for Individual Patients with Inflammatory Bowel Diseases. Clin. Gastroenterol. Hepatol..

[118] Staley C., Weingarden A.R., Khoruts A., Sadowsky M.J. (2017). Interaction of gut microbiota with bile acid metabolism and its influence on disease states. Appl. Microbiol. Biotechnol..

[119] Das P., Marcišauskas S., Ji B., Nielsen J. (2019). Metagenomic analysis of bile salt biotransformation in the human gut microbiome. BMC Genomics.

[120] Heinken A., Ravcheev D.A., Baldini F., Heirendt L., Fleming R.M.T., Thiele I. (2019). Systematic assessment of secondary bile acid metabolism in gut microbes reveals distinct metabolic capabilities in inflammatory bowel disease. Microbiome.

[121] Ringel Y., Chou C.J., Bruce S.J., Henry H., Lauber C., Betrisey B., Goun E., Ringel-Kulka T. (2019). Reduced Bacteria-Derived Bile Salt Hydrolase Enzymatic Activity—A Possible Mechanism for Symptoms Severity in Patients with Irritable Bowel Syndrome. Gastroenterology.

[122] Duboc H., Rajca S., Rainteau D., Benarous D., Maubert M.A., Quervain E., Thomas G., Barbu V., Humbert L., Despras G. (2013). Connecting dysbiosis, bile-acid dysmetabolism and gut inflammation in inflammatory bowel diseases. Gut.

[123] Chiang J.Y.L. (2017). Bile acid metabolism and signaling in liver disease and therapy. Liver Res..

[124] Ovadia C., Perdones-Montero A., Fan H.M., Mullish B.H., McDonald J., Papacleovoulou G., Wahlström A., Ståhlman M., Tsakmaki A., Clarke L. (2020). Ursodeoxycholic acid enriches intestinal bile salt hydrolase-expressing Bacteroidetes in cholestatic pregnancy. Sci. Rep..

[125] Manna L.B., Ovadia C., Lövgren-Sandblom A., Chambers J., Begum S., Seed P., Walker I., Chappell L.C., Marschall H.U., Williamson C. (2019). Enzymatic quantification of total serum bile acids as a monitoring strategy for women with intrahepatic cholestasis of pregnancy receiving ursodeoxycholic acid treatment: A cohort study. BJOG Int. J. Obstet. Gynaecol..

[126] Tabibian J.H., O’Hara S.P., Trussoni C.E., Tietz P.S., Splinter P.L., Mounajjed T., Hagey L.R., LaRusso N.F. (2016). Absence of the intestinal microbiota exacerbates hepatobiliary disease in a murine model of primary sclerosing cholangitis. Hepatology.

